# Fred Lorey Passed Away

**DOI:** 10.3390/ijns6040087

**Published:** 2020-11-04

**Authors:** Lisa Feuchtbaum, Michele A. Lloyd-Puryear

**Affiliations:** 1 Genetic Disease Screening Program, California Department of Public Health, 850 Marina Bay Parkway, F-175, MS 8200, Richmond, CA 94804, USA; 2 American College of Medical Genetics and Genomics, Bethesda, MD 20814, USA

To tackle the ever-increasing ambitions of the *International Journal of Neonatal Screening* (*IJNS*), in November 2019, we were looking for an Associate Editor to strengthen the Editorial board of *IJNS*. One obvious choice was Fred Lorey, who, with decades of experience in all aspects of newborn screening was a perfect candidate (an excellent overview of his persona and achievements is given by Lisa Feuchtbaum and Michele A. Lloyd-Puryear, below). Besides, we all knew Fred as a great colleague with an open mind and exactly that combination of an extensive network, original ideas, and the broad spectrum of knowledge concerning every aspect of neonatal screening to be able to generate the subjects that *IJNS* would need to cover, the critical view to discern which papers are valuable enough to be accepted in *IJNS*, and the ability to assist in editing when needed. We were happy to have a team with Fred, Can Figlioglu and Ralph Fingerhut and were looking ahead to a number of fruitful years for *IJNS*, only to be stopped short by Fred’s untimely passing. Fred died too young, he had just started his work for *IJNS* and in the short period that was granted him in his position, he already accomplished a lot. We are going to miss Fred tremendously.

On behalf of the Editorial Board and the Editorial management of

the *International Journal of Neonatal Screening*,

Ralph Fingerhut, Peter Schielen, Can Ficicioglu, Neil Ding

 

Dr. Fred Lorey (Figure 1) died unexpectedly, but peacefully, at home on 2 September 2020, with his brother, a friend, and his two Labrador Retrievers at his side. Fred was known world-wide as a pioneer in the field of genetic disease testing, and especially newborn screening, where his wide-ranging impact has undoubtedly improved the quality of life of countless newborns and their families in California and beyond. Fred contributed to over 70 publications throughout his professional career and was cited over 1600 times in the medical literature.

Dr. Lorey earned his PhD degree in Biological Anthropology and Genetics from the University of California at Davis where he studied albumin variants and bilirubin binding. After a professorship at the University of Minnesota, he accepted a position with the California Department of Public Health, Genetic Disease Screening Program (GDSP) where he worked for 25 years on program administration, research, development and evaluation. During his last four years at the GDSP, before his retirement, he served as the Acting Division Chief and his dedication to the delivery of the California Newborn Screening Program was unmatched. He oversaw the expansion of hemoglobinopathy screening to include Alpha Thalassemia in 1999. In 2005, he was instrumental in a major program expansion including disorders detected through tandem mass spectrometry and the addition of screening for congenital adrenal hyperplasia. In 2007, Fred oversaw the addition of cystic fibrosis and biotinidase deficiency, and in 2012, he worked with the Jeffrey Modell Foundation to implement newborn screening for Severe Combined Immunodeficiency (SCID) in California. The California Prenatal Screening Program also expanded under his leadership to include screening for chromosomal abnormalities in the first and second trimesters of pregnancy. In addition, Fred was instrumental in implementing the California Biobank Program that provides both newborn and prenatal screening specimens for approved research investigations. The newborn screening community and GDSP staff are mourning his loss. GDSP staff recognize the many contributions he made to the California program, staff, vision and culture. But he also left his mark internationally on the broader world of newborn screening.

On 29 October 2014 at the Newborn Screening Genetics Testing Symposium, the Association of Public Health Laboratories honored Fred with the prestigious George Cunningham Visionary Award in Newborn Screening for his expertise and commitment to newborn screening services. In a reflection on his death, Dr. Cunningham noted that he was really proud that Fred received this award and stated that, *“…he was an amazing warm and talented human being... I enjoyed working with him and the public benefited tremendously from his contributions and dedication. He lived a full life with many friends, and he will be missed.*”

After leaving the GDSP, Fred was a consultant in developing countries with PerkinElmer Life Sciences, where he worked on newborn screening program development and expansion in Tanzania, Morocco, India, Indonesia, Vietnam, China, Colombia, Mexico and Guatemala. Travelling with Fred at these international newborn screening meetings was always a joy and resulted in many memorable experiences.

Dr. Lorey was active in many organizations and served on many national, international and state advisory committees. He worked tirelessly to create and translate the tools needed to realize the promise of early diagnosis and treatment to save lives. Fred was an inaugural member of the Newborn Screening Translational Research Network (NBSTRN) Steering Committee and set the course for over a decade of facilitating ground-breaking research. Fred was a key contributor to a model project that piloted screening for SCID, the uniformly fatal immune deficiency. His efforts undoubtedly led to thousands of newborns and families having a chance for a healthy life. “*His research in newborn screening has benefitted babies widely*”, said Dr. Howell, the chair of the first NBSTRN steering committee. He was a member of the Society of Inherited Metabolic Disorders, the Association of Public Health Laboratories and the International Society of Neonatal Screening (ISNS), where he was previously the North American Council representative. Most recently he was serving on the editorial board of the ISNS’s *International Journal of Neonatal Screening*. Dr. Lorey also served for five years on the federal Advisory Committee for Heritable Disorders in Newborns and Children, which is the federal committee that advises the United States government on screening for heritable disorders for newborns and children. In his role on the Advisory Committee, Dr. Alex Kemper noted that, “*Fred was proud of the California newborn screening program and worked tirelessly to always improve it. He was committed to assuring that the newborn screening system worked to improve the lives of children. He was active with the Advisory Committee and focused on assuring that the Committee’s recommendations were based on science while recognizing that the recommendations could have a profound and direct impact on families.*” His work with families was exemplified by his work with the Save Babies Through Screening Foundation (SBTS), where he was a medical advisor for over 10 years and had recently joined their Board of Directors. Jill Levy-Fisch, of SBTS, stated “*Fred was an integral part of SBTS and gave a tremendous amount of time to further our work to protect the lives of newborns and children.*”

## Figures and Tables

**Figure 1:**
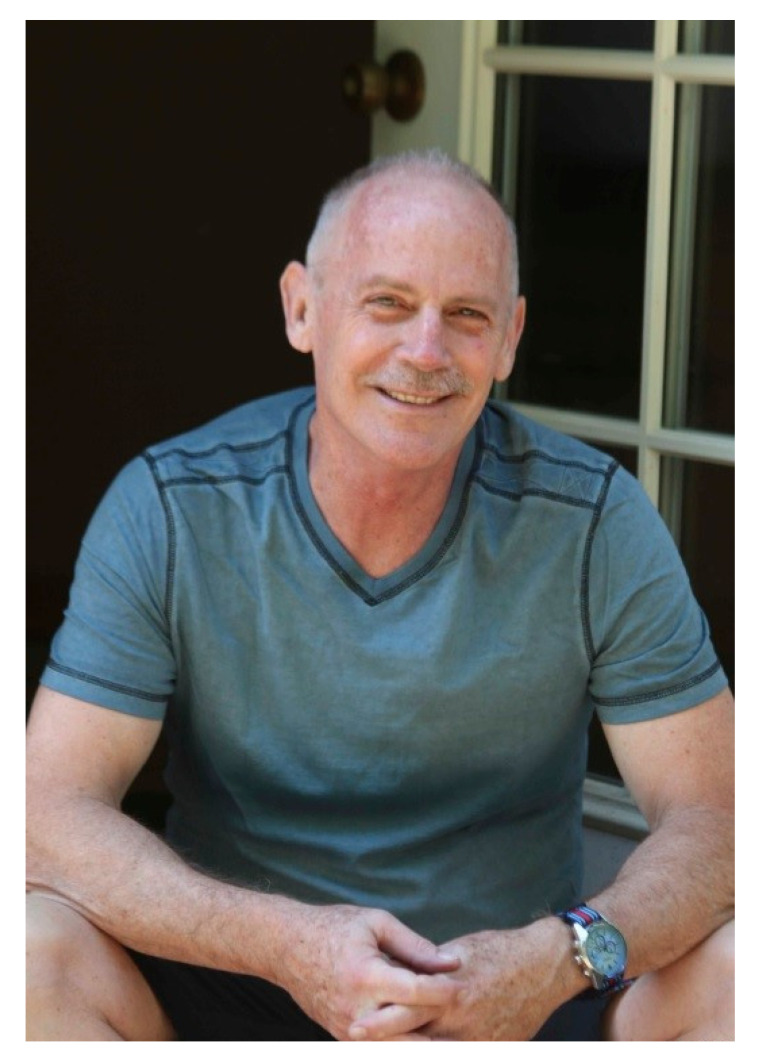
Recent picture of Dr. Fred Lorey (This photo was received from Dr. Fred Lorey's family with their permision to use it here).

